# Dietary Intake of Anti-Oxidant Vitamins A, C, and E Is Inversely Associated with Adverse Cardiovascular Outcomes in Chinese—A 22-Years Population-Based Prospective Study

**DOI:** 10.3390/nu10111664

**Published:** 2018-11-04

**Authors:** Chi-Ho Lee, Ruth S. M. Chan, Helen Y. L. Wan, Yu-Cho Woo, Chloe Y. Y. Cheung, Carol H. Y. Fong, Bernard M. Y. Cheung, Tai-Hing Lam, Edward Janus, Jean Woo, Karen S. L. Lam

**Affiliations:** 1 Department of Medicine, University of Hong Kong, Hong Kong, China; leechihopaul@gmail.com (C.-H.L.); wanyl@hku.hk (H.Y.L.W.); wooyucho@gmail.com (Y.-C.W.); cyy0219@hku.hk (C.Y.Y.C.); kalofong@gmail.com (C.H.Y.F.); mycheung@hku.hk (B.M.Y.C.); 2 Research Center of Heart, Brain, Hormone and Healthy Aging, University of Hong Kong, Hong Kong, China; 3 Department of Medicine and Therapeutics, The Chinese University of Hong Kong, Hong Kong, China; ruthchansm@cuhk.edu.hk; 4 The School of Public Health, University of Hong Kong, Hong Kong, China; hrmrlth@hkucc.hku.hk; 5 Department of Medicine-Western Health, Melbourne Medical School, The University of Melbourne, Melbourne, Victoria 3021, Australia; januse@netspace.net.au; 6 General Medical Unit, Western Health, St Albans, Victoria 3021, Australia

**Keywords:** anti-oxidant, vitamin A, vitamin C, vitamin E, adverse cardiovascular outcomes, chinese, prediction model

## Abstract

**Background:** Conflicting and population-dependent findings have been reported from epidemiological studies on the associations of dietary intake of anti-oxidant vitamins with cardiovascular events. We investigated the prospective relationship between dietary intake of anti-oxidant vitamins and incident adverse cardiovascular outcomes amongst Hong Kong Chinese. **Methods:** In this prospective population-based study, baseline dietary intake of anti-oxidant vitamins (A, C, and E) were assessed using a food frequency questionnaire in 875 Chinese participants from the Hong Kong Cardiovascular Risk Factor Prevalence Study (CRISPS) in 1995–1996. The adjusted hazard ratio (HR) of incident adverse cardiovascular outcomes, defined as the first recorded diagnosis of cardiovascular deaths, non-fatal myocardial infarction or non-fatal stroke, and coronary or other arterial revascularizations, was calculated per unit intake of each vitamin using multivariable Cox regression. **Results:** Over a median follow-up of 22 years, 85 participants (9.7%) developed adverse cardiovascular outcomes. Dietary intakes of vitamin A, C, and E were independently and inversely associated with incident adverse cardiovascular outcomes (HR 0.68, 95%CI 0.53–0.88, *p* = 0.003 for vitamin A; HR 0.66, 95%CI 0.52–0.85, *p* = 0.001 for vitamin C; and HR 0.57, 95%CI 0.38–0.86, *p* = 0.017 for vitamin E) after adjustments for conventional cardiovascular risk factors at baseline. **Conclusions:** Dietary intakes of anti-oxidant vitamins A, C, and E reduced the risk of adverse cardiovascular outcomes in Hong Kong Chinese.

## 1. Introduction

Cardiovascular disease (CVD) is a significant global health burden [1,2]. Strikingly, despite an overall decline in the age-standardized prevalence of CVD especially in high-income countries, cardiovascular mortality still accounted for a third of all deaths in 2015 [1]. Oxidative stress plays an important role in atherosclerosis, amidst the multiple common drivers of CVD progression [3,4], and anti-oxidant vitamins have been associated with reduced cardiovascular risk. Although supplementation with anti-oxidant vitamins overall did not show a reduction of incident major cardiovascular outcomes (cardiovascular death, fatal or non-fatal myocardial infarction, stroke, or transient ischemic attack) in randomized controlled trials [5,6], preclinical studies have consistently demonstrated beneficial effects on atherosclerosis, mainly through the attenuation of lipid peroxidation and free radical induced damage, of anti-oxidant vitamins A [7], C [8], and E [9,10]. Moreover, epidemiological studies have reported an inverse association between CVD and dietary intake of anti-oxidant vitamins A [11,12,13], C [13,14], and E [15,16,17,18]—although the relationships between the individual anti-oxidant vitamin and CVD are controversial and might vary depending on the study populations, which has been postulated to be explained in part by differences in their usual diet and baseline nutritional status [12,13,14,19]. Therefore, we investigated the relationship between dietary intake of anti-oxidant vitamins and incident adverse cardiovascular outcomes amongst Hong Kong Chinese, using a prospective population-based study with 22 years of follow-up.

## 2. Materials and Methods

### 2.1. Participants

All participants were recruited from the Hong Kong Cardiovascular Risk Factor Prevalence Study (CRISPS), which was the first population-based study with comprehensive cardiovascular risk assessment in Hong Kong Chinese [20]. In 1995–1996 (CRISPS-1), 2900 individuals, aged 25 to 74, were recruited from the general population through random selection of telephone numbers, with at least 200 individuals included in each 10-year age group. All subjects provided written informed consent before their participation in the study. The study protocol was approved by the ethics committee of the University of Hong Kong (EC 849-96), and the clinical research committee of the Chinese University of Hong Kong.

Participants at CRISPS-1 attended a health assessment in our hospital after an overnight fast of 12 h to determine whether they had major cardiovascular risk factors, including obesity, type 2 diabetes, hypertension and dyslipidemia. Using a detailed questionnaire, demographic data, which included age, gender, smoking, alcohol consumption and physical activity, and medical, drug and family histories were obtained. Anthropometric parameters, including body weight, height, body mass index (BMI), waist circumference (WC) and blood pressure were measured. Unless participants were on anti-diabetic medications, all had a 75-g oral glucose tolerance test (OGTT) with blood also drawn for fasting lipid profile and serum insulin. All participants from CRISPS-1 were then contacted and invited for subsequent prospective follow-up visits (CRISPS-2 in 2000-04, *N* = 1944; CRISPS-3 in 2005-08, *N* = 1802 and CRISPS-4 in 2010-12, *N* = 1618). 

**Figure 1:**
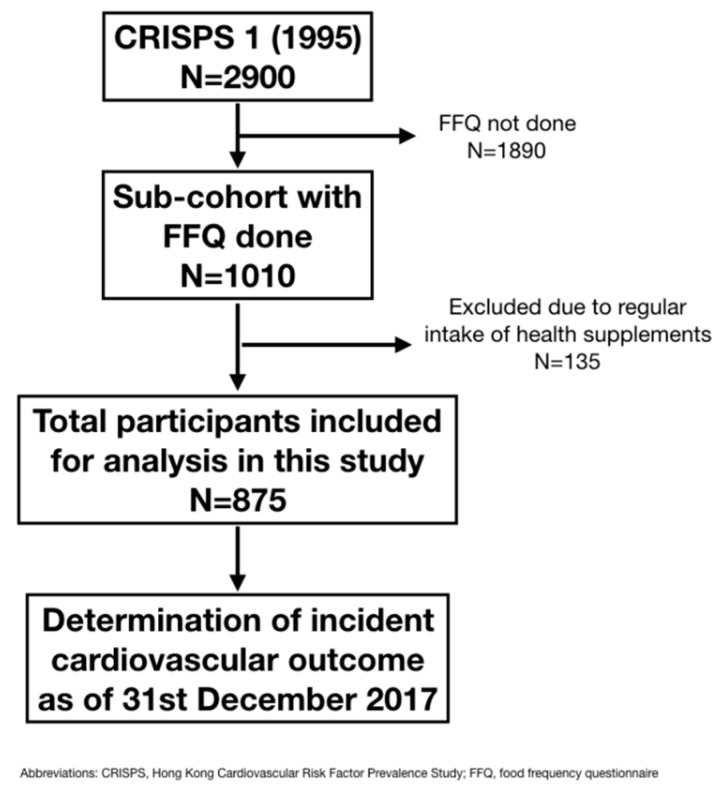
Flowchart describing the study participants. CRISPS, Cardiovascular Risk Factor Prevalence Study; FFQ, food frequency questionnaire.

In this paper on the association between dietary intake of antioxidant vitamins and incident adverse cardiovascular outcomes, we employed data from a sub-cohort in CRISPS-1 consisting of 1010 participants (499 men and 511 women) who consented to this sub-study, which was conducted in the first year of CRISPS-1 (Figure 1). Dietary assessment was performed consecutively on those who attended, until at least 100 participants in each 10-year age and sex groups from <35 years, 35–44 years, 45–54 years and >54 years were recruited. Their detailed dietary history was taken using a food frequency questionnaire with 7-day recall as described previously [21,22]. Using food composition tables for Hong Kong, quantification of each nutrient intake was derived by summation of the nutrients obtained from all food items in the food frequency questionnaire. Since sodium and potassium intake derived from food composition tables were much lower than expected, urinary sodium and potassium measurements were used to estimate dietary intake of sodium and potassium in our study participants as previously described [21]. In this study, participants who reported regular intake of health supplements at baseline were excluded for analysis.

### 2.2. Definitions of Clinical Variables

Hypertension was defined as blood pressure ≥140/90 mmHg or on anti-hypertensive medications. Dyslipidemia was defined as fasting triglycerides (TG) ≥ 1.69 mmol/L, high-density lipoprotein cholesterol (HDL-C) < 1.04 mmol/L in men and <1.29 mmol/L in women, low-density lipoprotein cholesterol (LDL-C) ≥ 3.4 mmol/L or on lipid lowering agents. For glycemic status, since glycated hemoglobin (HbA1c) was not measured at CRISPS-1, type 2 diabetes was defined according to the World Health Organization (WHO) 1998 diagnostic criteria: Fasting glucose (FG) ≥ 7 mmol/L or 2-h post OGTT glucose (2hG) ≥ 11.1 mmol/L, or on anti-diabetic medications [23]. Dysglycemia was defined as impaired fasting glucose with FG ≥ 6.0 mmol/L, impaired glucose tolerance with 2hG ≥ 7.8 mmol/L or the presence of type 2 diabetes [24].

### 2.3. Cardiovascular Outcomes

Adverse cardiovascular outcomes, the outcome of interest in our study, were defined as the first recorded diagnosis of hard cardiovascular endpoints, including cardiovascular deaths, non-fatal myocardial infarction or non-fatal stroke, and coronary or other arterial revascularizations as of 31 December 2017. At baseline (CRISPS-1) and each follow-up visits (CRISPS-2-4), the occurrence of CVD and other health conditions were determined from both the questionnaire and health assessments, with verification from the Clinical Management System (CMS) of the Hospital Authority using the ninth edition of the International Codes of Diagnosis (ICD-9) codes, as well as from their private practitioners. Death events were retrieved from the Hong Kong Death Registry. Moreover, for those participants whose baseline data at CRISPS-1 were available, but did not return for subsequent follow-up visits, their relevant clinical information was traced from the CMS of the Hospital Authority. All outcome events were adjudicated by two physicians reviewing the case records independently.

### 2.4. Statistical Analysis

All analyses were performed with IBM SPSS Statistics 24. Clinical variables that were not normally distributed, as determined using Kolmogorov-Smirnov test, were natural-logarithmically transformed to obtain near normality before analysis. All nutrient variables were also log-base2 transformed before analysis. Values were reported as means ± standard deviation (SD) or medians with inter-quartile range (IQR) as appropriate. All nutrient intake were adjusted for total energy intake using the residual method [25]. Multivariable Cox regression analysis was used to examine the associations of baseline dietary intake of anti-oxidant vitamins A, C, and E, with development of adverse cardiovascular outcomes. The variables included in Cox regression models were those that were either statistically or biologically significant. The hazard ratio (HR) for vitamins A, C, and E referred to the risk of adverse cardiovascular outcomes per unit increase in the log-transformed, or a doubling of daily intake of each anti-oxidant vitamin measured in IU for vitamin A, and mg for vitamin C and E. In all statistical tests, two-sided *p*-values < 0.05 were considered significant.

## 3. Results

Among the 2900 participants recruited in CRISPS-1, 1010 participants had dietary data. Participants who had food frequency questionnaire performed were not significantly different, with regard to their baseline clinical characteristics, from those who did not. (Supplemental Table S1). After excluding 135 participants who took health supplements at least once weekly at baseline, a total of 875 participants (456 men and 419 women) were included in the analysis. Table 1 summarizes their baseline characteristics. The mean age of our study participants was 44.7 ± 11.5 years old with a mean BMI and WC of 24.3 ± 3.63 kg/m^2^ and 79.8 ± 10.2 cm, respectively. Among them, 25.5% were smokers, 9.7%, 61.4% and 2.6% of them had dysglycemia, dyslipidemia and history of cardiovascular disease at baseline, respectively. Over a median follow-up of 22 years, 85 participants (9.7%) developed adverse cardiovascular outcomes. Those who had incident cardiovascular events were more likely men (*p* < 0.001), smokers (*p* < 0.001), and were significantly older (*p* < 0.001) with higher BMI (*p* = 0.005), WC (*p* < 0.001), systolic blood pressure (*p* < 0.001), FG (*p* < 0.001), 2hG (*p* < 0.001), LDL-C (*p* < 0.001), but lower HDL-C (*p* = 0.003), compared to those who did not. Moreover, there was a significantly higher prevalence of dysglycemia (*p* = 0.025), dyslipidemia (*p* = 0.001) and CVD (*p* = 0.007) at baseline among those who developed adverse cardiovascular outcomes compared to those who did not. However, both urinary sodium and potassium concentrations, which reflected estimates of dietary intake of sodium and potassium, respectively, were not significantly different between those with and without incident adverse cardiovascular outcomes. 

Table 2 summarizes the baseline nutrient intake of the study participants. Compared to those who did not develop adverse cardiovascular outcomes, participants who had incident cardiovascular events had lower intake of anti-oxidant vitamins A and C in both men and women. There were similar but much smaller differences for vitamin E.

In multivariable Cox regression analysis (Table 3), all three anti-oxidant vitamins A, C, and E were significantly associated with incident adverse cardiovascular outcomes after adjustments for age and sex (HR 0.67, 95%CI 0.53–0.86, *p* = 0.002 per IU of vitamin A; HR 0.66, 95%CI 0.51–0.84, *p* = 0.001 per mg of vitamin C; and HR 0.60, 95%CI 0.41–0.90, *p* = 0.012 per mg of vitamin E). Upon further adjustments for BMI, smoking, hypertension, dyslipidemia, dysglycemia and history of CVD at baseline, dietary intake of anti-oxidants A, C, and E remained independent predictors of long-term development of adverse cardiovascular outcomes (HR 0.68, 95%CI 0.53–0.88, *p* = 0.003 for vitamin A; HR 0.66, 95%CI 0.52–0.85, *p* = 0.001 for vitamin C; and HR 0.57, 95%CI 0.38–0.86, *p* = 0.001 for vitamin E). In the three multivariable Cox regression models consisting of the above cardiovascular risk factors and either vitamin A, C, or E, age (HR 1.07), hypertension (HR 1.75–1.79) and smoking (HR 2.22–2.29) were the other independent predictors of incident adverse cardiovascular outcomes. Sex interaction was not present in vitamins A, C, and E intake.

Dietary intake of anti-oxidants A, C, and E were highly correlated with fiber intake (*r* >0.6 in both men and women), and women had significantly higher intake of vegetables (*p* = 0.029) and fruits (*p* < 0.001) than men in our study. However, using the backward elimination method, increasing quartiles of dietary intake of vitamins A, C, and E remained independently associated with reduced adverse cardiovascular outcomes with no substantial differences before and after adjustment for fiber intake (*p* for trend with adjustment: 0.014, 0.002 and 0.046, respectively) (Table 4).

**Figure 2:**
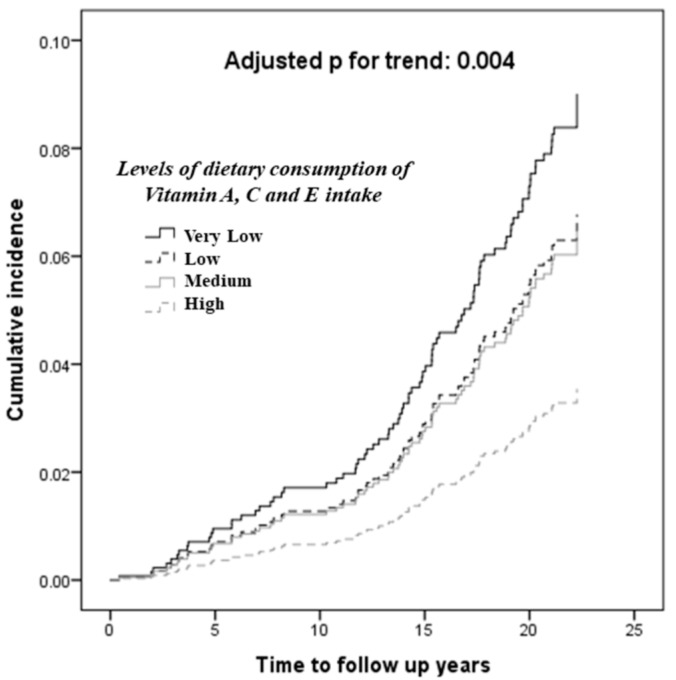
The association of increasing dietary intake of vitamins A, C, and E with adverse cardiovascular outcomes. The 4 groups, “very low”, “low”, “medium” and “high” intake, were defined as having 0, 1, 2 and 3 of the anti-oxidant vitamins being above median, respectively. The above model was adjusted for gender, age, body mass index, ever-smoking, hypertension, dyslipidemia, dysglycemia and history of cardiovascular disease at baseline.

Furthermore, Figure 2 shows that when the study participants were classified into 4 groups according to their levels of daily intake of vitamins A, C, and E, as “very low”, “low”, “medium”, and “high” intake, defined as having, respectively, 0, 1, 2, and 3 of the anti-oxidant vitamins being above median, an increasing risk of incident adverse cardiovascular outcomes, after adjustments for sex, age, BMI, ever-smoking, hypertension, dyslipidemia, dysglycemia, and history of CVD at baseline (adjusted p for trend <0.001), and the association remaining significant after further adjustments for fiber intake. (*p* for trend after further adjusted for fiber = 0.038).

## 4. Discussion

To our knowledge, the current study, which has shown the inverse associations between dietary intake of anti-oxidant vitamins and incident fatal and non-fatal CVD in Chinese, is one of the longest prospective population-based studies in addressing such associations in any population. We demonstrated that low dietary intake of vitamins A, C, and E all independently predicted lower risk of adverse cardiovascular outcomes in Chinese, a very large subset of the human population, above and beyond conventional risk factors of cardiovascular diseases.

Previous epidemiological findings on the relationship between dietary intake of anti-oxidant vitamins and adverse cardiovascular outcomes were conflicting and seemed to vary geographically. For instance, while dietary intake of vitamin A was found to be inversely associated with coronary artery disease in a few studies performed in the United States [11,16,26] and the Netherlands [12], no association with coronary artery disease and CVD was found in studies conducted in Finland [17] and Japan [14,27], respectively. In contrast, low dietary intake of vitamin C was associated with increased risk of CVD in women [14], as well as stroke among non-smokers in Japan [27]. However, no clear association was reported in either the American [11,15,16,18] or Dutch studies [12], although a trend was noted in the Finnish cohort [17]. Similar studies on Chinese are scarce. A recent study using two prospective cohorts in Shanghai, which examined the associations between dietary intake of anti-oxidant vitamins and mortality outcomes, found that dietary intakes of vitamins A and C were inversely associated with all-cause and cardiovascular mortality [13]. 

Several reasons had been proposed to account for these inconsistent epidemiological observations, which included differences in dietary habits, food source, background nutritional status and clinical characteristics of the study participants, as well as variations in the adjustments for confounders [13,19]. In fact, the authors of the Shanghai study had also attributed their lack of association between dietary vitamin E intake and cardiovascular mortality, at least in part, to the baseline nutritional status of their participants. Indeed, as compared with ours, their median dietary vitamin E intake was unequivocally closer to the daily recommended dietary allowance (RDA) of 15 mg for vitamin E [28] (9.88 mg/day in our cohort vs. 14.56 mg/day and 13.12 mg/day in Shanghai cohorts). Therefore, it was not surprising that additional dietary vitamin E intake in the Shanghai cohorts might not provide as much beneficial cardiovascular effects as it did in our study participants who had relatively low vitamin E level. 

Our findings also highlighted the cardiovascular effects of more than adequate dietary intake of vitamins A and C. Previous randomized controlled trials failed to demonstrate benefits, or even harm, with anti-oxidant vitamin supplementation [5]. Moreover, a U-shaped relationship between serum vitamin A levels and cardiovascular mortality among adults in the United States has been reported previously [29]. The RDA in men and women are 2330 IU and 3000 IU for vitamin A in retinol, and 75 mg and 90 mg for vitamin C, respectively. Therefore, in the current study, contrary to that of vitamin E, the median daily intake of both vitamins A and C of our study participants were at least close to, if not more than the RDA of both vitamins A and C, with some participants having intake of vitamins A and C at as high as 5000 IU and 140 mg, respectively [21]. Despite this, our study provided observational evidence that such higher than adequate intake of vitamins A and C, through diet but not supplements, not only did not increase cardiovascular events, but showed lower risk of developing adverse cardiovascular outcomes after a median follow-up of 22 years. 

In fact, vitamin supplements should not be perceived as entirely equivalent to anti-oxidant vitamins obtained from food [30]. This is analogous to the recommendations of eating foods rich in long-chain n-3 fatty acids in diabetes, but not supporting the routine n-3 supplements based on findings from randomized controlled trials [31]. Fruits and vegetables are the main food source for these anti-oxidant vitamins especially vitamin C [32,33]. In the current study, although the beneficial effects of these anti-oxidant vitamins were not observed secondary to the fiber content of the fruits and vegetables, it was possible that part of these long term cardiovascular benefits might come from other micronutrients (e.g., polyphenols), which share the same food source with vitamins A, C, and E. Since these food source also varies among individuals from different populations, therefore, these dietary effects might not be easily replicated by randomized controlled trials using vitamin supplements and placebo. 

Our study has several limitations. Firstly, the small number of events especially in women limited further subgroup analyses on gender-specific effects, and the associations of anti-oxidant vitamins with each individual cardiovascular outcome. Secondly, baseline circulating vitamin levels were not available in our study participants. However, dietary intake was known to correlate poorly with circulating vitamin levels especially for vitamins C and E [27,34]. Thirdly, nutrient data was not available in subsequent follow-up visits, so that changes in vitamin intake could have occurred over the years, which would have attenuated the associations with baseline data. Furthermore, reverse causality could potentially occur in all observational studies, such that participants who had CVD might already have attempted to eat more healthily in the run up to their events. However, this is less likely to happen in our study with such a long duration of follow-up. Last, but not least, residual confounding might still be possible as data on some factors, for example the absolute levels of physical activity and alcohol consumption etc. that could also impact on cardiovascular risk, was not available, due to limitations in the initial cohort design. Nonetheless, the 22-year of median follow-up in a well-designed prospective cohort representative of the general population of Hong Kong Chinese is certainly a major strength among similar epidemiological studies.

## 5. Conclusions

Fruits and vegetables are food source rich in anti-oxidant vitamins. However, despite recent evidence showing that high intake of fruits, vegetables and legumes also reduced the risks of non-cardiovascular and total mortality [35], the mean intake of fruit and vegetables are still lower than current recommendations globally [36,37]. Our study demonstrated that low dietary intake of anti-oxidant vitamins A, C, and E all significantly increased the long-term risk of developing adverse cardiovascular outcomes in Hong Kong Chinese. Increasing intake of each of these antioxidant vitamins was associated with a modest, but clinically significant, risk reduction. This may have significant implications for the larger worldwide Chinese community. Our findings suggest that measures to promote adequate intake of these vitamins, from fruits and vegetables, as well as other food sources, such as nuts, seeds and dairy products, should form part of the public health policy to reduce the morbidity and mortality due to cardiovascular diseases.

## Figures and Tables

**Table 1:** Baseline characteristics of study participants stratified by adverse cardiovascular events.

Baseline Variables	All	Adverse Cardiovascular Events		
		No	Yes	*p*-Value
N, %	875	790	85	--
Age, years	44.7 ± 11.5	43.7 ± 10.1	54.1 ± 11.5	**<0.001**
Men, %	52.1	49.7	74.1	**<0.001**
Ever smokers, %	25.5	22.7	51.8	**<0.001**
BMI, kg/m^2^	24.3 ± 3.63	24.2 ± 3.61	25.4 ± 3.69	**0.005**
Waist circumference, cm				
Men	83.2 ± 9.46	82.6 ± 9.16	87.2 ± 10.4	**<0.001**
Women	76.0 ± 9.70	75.6 ± 9.61	82.9 ± 8.83	**0.001**
Hypertension, %	17.3	14.8	40.0	**<0.001**
Systolic BP, mmHg	119 ± 18.5	118 ± 17.8	130 ± 21.1	**<0.001**
Diastolic BP, mmHg	75.0 ± 11.0	74.4 ± 10.7	80.6 ± 12.0	**<0.001**
Dysglycemia, %	9.7	7.1	14.1	**0.025**
Fasting glucose, mmol/L	5.36 ± 1.15	5.30 ± 0.99	5.88 ± 2.06	**<0.001**
2-h glucose, mmol/L	6.65 ± 3.21	6.53 ± 2.95	7.72 ± 4.87	**0.001**
Dyslipidemia, %	61.4	59.6	77.6	**0.001**
HDL-C, mmol/L	1.28 ± 0.36	1.29 ± 0.35	1.18 ± 0.30	**0.003**
LDL-C, mmol/L	3.26 ± 0.87	3.21 ± 0.84	3.73 ± 0.96	**<0.001**
Triglycerides *, mmol/L	1.0 (0.70–1.40)	1.0 (0.70–1.40)	1.1 (0.90–1.56)	**0.009**
History of CVD at baseline, %	2.6	2.2	7.1	**0.007**
Urinary sodium *, mg/day	3953 (2494–5566)	3958 (2531–5558)	3884 (2386–6080)	0.612
Urinary potassium *, mg/day	2523 (1941–3488)	2629 (1967–3518)	2466 (1733–3418)	0.389

*, Logarithmically-transformed before analysis; Values in **BOLD** were statistically significant. Hypertension was defined as blood pressure ≥140/90 mmHg or on anti-hypertensive medications; Dyslipidemia was defined as TG ≥ 1.69 mmol/L, HDL-C < 1.04 mmol/L in men and <1.29 mmol/L in women, LDL-C ≥ 3.4 mmol/L or on lipid-lowering agents; Dysglycemia was defined as any impaired fasting glucose, impaired glucose tolerance or type 2 diabetes based on World Health Organization criteria. Conversion factors for glucose from mmol/liter to mg/dL × 18; HDL/LDL-C from mmol/liter to mg/dL × 38.9; Triglyceride from mmol/liter to mg/dL × 88.2. BMI, body mass index; BP, blood pressure; HDL-C, high density lipoprotein-cholesterol; LDL-C, low density-lipoprotein cholesterol; CVD, cardiovascular disease.

**Table 2:** Baseline daily nutrient intake of study participants obtained by food frequency questionnaire, stratified by adverse cardiovascular events.

	All	Men		Women	
		Adverse Cardiovascular Events		Adverse Cardiovascular Events	
Baseline variables		No	Yes	No	Yes
N	875	393	63	397	22
Energy, kcal	2030 (1671–2481)	2535 (1983–2781)	2168 (1807–2584)	1743 (1481–2073)	1696 (1413–1916)
Protein, g	92.1 (73.2–118)	106 (84.5–132)	96.7 (75.1–117.7)	80.9 (65.2–101)	81.1 (61.4–103)
Fat, g	64.7 (49.2–83.1)	74.2 (59.2–117.7)	61.3 (48.8–89.1)	55.8 (44.4–70.4)	50.4 (40.8–64.3)
Carbohydrate, g/day	268 (221–335)	317 (261–369)	287 (244–377)	233 (191–278)	227 (198–256)
Vitamin A, IU/day	3907 (2584–5758)	3966 (2607–5711)	3411 (2070–5310)	4015 (2731–5987)	3117 (2044–5436)
Vitamin B1, mg/day	0.95 (0.72–1.22)	1.08 (0.85–1.38)	0.97 (0.75–1.35)	0.83 (0.66–1.06)	0.78 (0.64–0.65)
Vitamin B2, mg/day	1.05 (0.79–1.32)	1.15 (0.91–1.46)	1.03 (0.82–1.27)	0.95 (0.73–1.24)	0.91 (0.67–1.25)
Niacin, mg/day	16.8 (12.9–21.7)	19.0 (14.9–24.0)	16.9 (13.5–22.1)	14.8 (11.5–18.8)	14.5 (12.1–19.7)
Vitamin C, mg/day	141.7 (95.9–199)	132 (84.8–186)	116 (58.0–180)	153 (110–220)	129 (96.7–188)
Vitamin D, ug/day	10.1 (5.00–20.4)	13.0 (5.21–25.0)	12.4 (5.53–21.5)	9.00 (5.00–15.0)	6.06 (4.69–10.2)
Vitamin E, mg/day	9.88 (7.53–13.0)	10.0 (7.77–13.7)	9.91 (6.29–12.6)	9.65 (7.54–12.4)	9.64 (5.34–13.2)
Calcium, mg/day	537 (412–697)	558 (430–728)	517 (382–655)	529 (398–691)	439 (348–671)
Phosphorus, mg/day	1078 (853–1322)	1217 (992–1459)	1101 (883–1299)	954 (772–1139)	925 (703–1100)
Iron, mg/day	15.3 (11.9–19.0)	16.6 (13.2–20.6)	15.1 (11.7–18.8)	13.7 (11.0–17.7)	14.2 (10.1–18.3)
Zinc, mg/day	11.2 (8.84–14.6)	13.0 (10.7–16.4)	12.3 (9.21–15.6)	9.63 (7.88–12.0)	10.0 (6.95–12.5)
Iodine, ug/day	0.21 (0.00–0.61)	0.21 (0.00–0.71)	0.04 (0.00–0.55)	0.21 (0.00–0.66)	0.16 (0.00–0.32)
Copper, mg/day	12.1 (8.90–15.5)	12.9 (9.67–16.3)	10.1 (8.0.–15.3)	11.4 (8.85–14.5)	10.0 (7.11–13.4)
Fibre, g/day	7.54 (5.61–9.98)	7.29 (5.236–9.88)	6.79 (4.33–9.57)	7.92 (6.09–10.1)	6.21 (4.74–9.07)
SFA, g/day	17.2 (12.7–23.2)	20.6 (15.5–26.5)	17.7 (12.5–26.3)	15.1 (11.1–19.8)	14.0 (11.0–17.0)
MUFA, g/day	21.8 (16.5–29.8)	26.3 (20.1–33.8)	22.0 (16.1–32.7)	19.3 (14.6–24.1)	18.3 (14.1–21.3)
PUFA, g/day	14.4 (11.3–18.1)	16.1 (12.8–20.2)	13.7 (11.1–18.7)	12.8 (10.7–15.6)	11.7 (8.02–15.7)
Cholesterol, mg/day	307 (225–426)	368 (263–505)	343 (226–450)	267 (193–363)	240 (178–278)
Protein, % energy/day	18.6 ± 3.12	18.4 ± 3.00	17.8 ± 2.87	18.8 ± 3.22	19.5 ± 3.36
Carbohydrate, % energy/day	53.5 ± 7.67	53.5 ± 7.60	54.9 ± 9.16	53.3 ± 7.53	53.3 ± 6.51
Fat, % energy/day	28.9 ± 5.58	28.7 ± 5.57	27.9 ± 6.97	29.2 ± 5.39	28.4 ± 4.18

Data were presented as mean ± standard deviation or median (25th–75th percentile) as appropriate. All variables were log-base2 transformed before analysis except the % of energy from protein, carbohydrate and fat. HR, hazard ratio; SFA, saturated fatty acid; MUFA, monounsaturated fatty acid; PUFA, polyunsaturated fatty acid.

**Table 3:** Multivariable Cox regression analysis on the associations between each unit increase of nutrient intake and adverse cardiovascular outcomes.

	Model 1		Model 2	
	Adjusted Hazard Ratio (95%CI)	*p*-Value	Adjusted Hazard Ratio (95%CI)	*p*-Value
Protein, g/day	1.29 (0.53–3.12)	0.57	1.13 (0.47–2.73)	0.79
Fat, g/day	1.34 (0.66–2.73)	0.42	1.30 (0.64–2.64)	0.47
Carbohydrate, g/day	0.48 (0.17–1.37)	0.17	0.57 (0.20–1.60)	0.29
Vitamin A, IU/day	**0.67 (0.53–0.86)**	**0.002**	**0.68 (0.53–0.88)**	**0.003**
Vitamin B1, mg/day	1.05 (0.58–1.89)	0.87	1.01 (0.56–1.84)	0.96
Vitamin B2, mg/day	0.75 (0.42–1.34)	0.34	0.64 (0.36–1.16)	0.14
Niacin, mg/day	1.21 (0.66–2.25)	0.54	1.08 (0.58–2.02)	0.80
Vitamin C, mg/day	**0.66 (0.51–0.84)**	**0.001**	**0.66 (0.52–0.85)**	**0.001**
Vitamin D, ug/day	1.10 (0.95–1.27)	0.20	1.07 (0.92–1.24)	0.36
Vitamin E, mg/day	**0.60 (0.41–0.90)**	**0.012**	**0.57 (0.38–0.86)**	**0.007**
Calcium, mg/day	0.71 (0.46–1.09)	0.11	0.71 (0.45–1.10)	0.12
Phosphorus, mg/day	0.93 (0.36–2.38)	0.88	0.81 (0.31–2.12)	0.66
Iron, mg/day	0.55 (0.29–1.07)	0.08	0.54 (0.28–1.06)	0.07
Zinc, mg/day	1.50 (0.87–2.59)	0.15	1.32 (0.77–2.25)	0.31
Iodine, ug/day	0.94 (0.65–1.37)	0.75	0.94 (0.64–1.37)	0.73
Copper, mg/day	**0.67 (0.51–0.89)**	**0.005**	**0.69 (0.52–0.91)**	**0.008**
Fiber, g/day	**0.52 (0.37–0.75)**	**<0.001**	**0.54 (0.37–0.77)**	**0.001**
SFA, g/day	1.64 (0.95–2.82)	0.08	1.69 (0.96–2.98)	0.07
MUFA, g/day	1.58 (0.87–2.85)	0.13	1.60 (0.87–2.94)	0.13
PUFA, g/day	0.85 (0.46–1.59)	0.62	0.86 (0.46–1.61)	0.64
Cholesterol, mg/day	1.41 (0.92–2.17)	0.11	1.40 (0.89–2.19)	0.14
Protein, % energy/day	1.02 (0.96–1.09)	0.54	1.01 (0.95–1.08)	0.73
Carbohydrate, % energy/day	0.98 (0.96–1.01)	0.15	0.98 (0.96–1.01)	0.19
Fat, % energy/day	1.02 (0.99–1.06)	0.21	1.02 (0.99–1.06)	0.22

Values in **BOLD** were statistically significant. All variables were log-base2 transformed and energy adjusted using residual method before analysis, except the % of energy from protein, carbohydrate and fat. Model 1, adjusted for age and gender; Model 2, adjusted for age, gender, body mass index, smoking, hypertension, dyslipidemia, dysglycemia and history of cardiovascular disease at baseline. Hypertension was defined as blood pressure ≥140/90 mmHg or on anti-hypertensive medications; Dyslipidemia was defined as TG ≥ 1.69 mmol/L, HDL-C < 1.04 mmol/L in men and <1.29 mmol/L in women, LDL-C ≥ 3.4 mmol/L or on lipid-lowering agents; Dysglycemia was defined as any impaired fasting glucose, impaired glucose tolerance or type 2 diabetes based on World Health Organization criteria. SFA, saturated fatty acid; MUFA, monounsaturated fatty acid; PUFA, polyunsaturated fatty acid; HDL-C, high density lipoprotein-cholesterol; LDL-C, low density-lipoprotein cholesterol; TG, triglyceride.

**Table 4:** Multivariable Cox regression analysis on the associations between quartiles of each anti-oxidant vitamin and adverse cardiovascular outcomes with and without adjustment for fiber intake.

		Q1 (Lowest Intake)	Q2	Q3	Q4 (Highest Intake)	*p*-Trend
Vitamin A, IU/day	Model 1	1.00	0.55 (0.31–0.97)	0.58 (0.32–1.07)	0.45 (0.24–0.83)	**0.014**
	Model 2	1.00	0.55 (0.31–0.97)	0.58 (0.32–1.06)	0.45 (0.25–0.84)	0.014 *
Vitamin C, mg/day	Model 1	1.00	1.13 (0.65–1.98)	0.69 (0.39–1.24)	0.37 (0.18–0.73)	**0.002**
	Model 2	1.00	1.16 (0.66–2.02)	0.71 (0.40–1.27)	0.38 (0.19–0.74)	0.002 *
Vitamin E, mg/day	Model 1	1.00	0.68 (0.38–1.19)	0.51 (0.28–0.93)	0.57 (0.31–1.04)	**0.035**
	Model 2	1.00	0.68 (0.39–1.20)	0.51 (0.28–0.94)	0.59 (0.33–1.08)	0.046 *

*, Backward elimination method was applied to avoid multicollinearity. All variables were energy adjusted using residual method. Values in **BOLD** were statistically significant. Model 1 included gender, age, BMI, smoking, hypertension, dyslipidemia, dysglycemia, history of CVD. Model 2 included gender, age, BMI, smoking, hypertension, dyslipidemia, dysglycemia, history of CVD and fiber intake. Hypertension was defined as blood pressure ≥140/90 mmHg or on anti-hypertensive medications; Dyslipidemia was defined as TG ≥ 1.69 mmol/L, HDL-C < 1.04 mmol/L in men and <1.29 mmol/L in women, LDL-C ≥ 3.4 mmol/L or on lipid-lowering agents; Dysglycemia was defined as any impaired fasting glucose, impaired glucose tolerance or type 2 diabetes based on World Health Organization criteria. BMI, body mass index; CVD, cardiovascular disease; HDL-C, high density lipoprotein-cholesterol; LDL-C, low density-lipoprotein cholesterol; TG, triglyceride.

## Supplementary Materials

The following are available online at http://www.mdpi.com/2072-6643/10/11/1664/s1, Table S1: Baseline characteristics of CRISPS study participants with and without food frequency questionnaire (FFQ) done.
