# Supplementary material for: Dietary Intake of Anti-Oxidant Vitamins A, C, and E Is Inversely Associated with Adverse Cardiovascular Outcomes in Chinese—A 22-Years Population-Based Prospective Study
Source: Nutrients. 2018 Nov 4;10(11):1664. doi: 10.3390/nu10111664 (PMC6265686; doi:10.3390/nu10111664)
Supplement: Supplementary file 1 [file nutrients-10-01664-s001.pdf]

Supplemental table 1. Baseline characteristics of CRISPS study participants with and without food frequency questionnaire (FFQ) done

Baseline variables	All (n=2900)	FFQ not done (n=1890)	FFQ done (n=1010)	p-value
N, %	2900	1890, 65.2	1010, 34.8	--
Age, years	45.7±12.8	46.0±13.3	45.0±11.7	**0.06**
Men, %	48.7	48.3	49.4	0.54
Ever smoker, %	25.5	26.3	24.1	0.19
BMI, kg/m^2	24.1±3.61	24.1±3.61	24.2±3.62	0.56
Waist circumference, cm				
Men	83.0±9.57	83.0±9.53	83.1±9.65	0.95
Women	75.3±9.39	75.1±9.37	75.7±9.43	0.24
Systolic BP, mmHg	119.3±19.8	119.3±20.5	119.3±18.4	0.91
Diastolic BP, mmHg	74.7±10.8	74.5±10.7	74.9±10.9	0.36
Fasting glucose, mmol/L	5.39±1.39	5.42±1.51	5.33±1.52	0.09
2-hour glucose, mmol/L	6.79±3.13	6.83±3.15	6.72±3.08	0.13
HDL-C, mmol/L	1.25±0.33	1.23±0.32	1.29±0.34	**<0.001**
LDL-C, mmol/L	3.26±0.87	3.26±0.87	3.27±0.88	0.82
Triglycerides*, mmol/L	1.00 (0.70-1.50)	1.00 (0.70-1.50)	1.00 (0.70-1.40)	**0.03**
Hypertension, %	18.1	18.5	17.4	0.26
Dyslipidemia, %	64.4	66.2	60.9	**0.002**
History of CVD at baseline, %	2.8	2.9	2.6	0.39

*Logarithmically-transformed before analysis; Values in **BOLD** were statistically significant.
Hypertension was defined as blood pressure ≥140/90 mmHg or on anti-hypertensive medications.
Dyslipidaemia was defined as TG ≥1.69 mmol/L, HDL-C <1.04 mmol/L in men and <1.29 mmol/L in women, LDL-C ≥3.4 mmol/L or on lipid-lowering agents
Conversion factors for glucose from mmol/liter to mg/dL x18; HDL / LDL-C from mmol/liter to mg/dL x38.9; Triglyceride from mmol/liter to mg/dL x88.2.
CRISPS, Cardiovascular Risk Factor Prevalence Study; FFQ, food frequency questionnaire; BMI, body mass index; BP, blood pressure; HDL-C, high density lipoprotein-cholesterol; LDL-C, low density-lipoprotein cholesterol; CVD, cardiovascular disease
